# A Study of Optimizing Lamb Wave Acoustic Mass Sensors’ Performance through Adjustment of the Transduction Electrode Metallization Ratio

**DOI:** 10.3390/s22176428

**Published:** 2022-08-26

**Authors:** Fatemeh Gholami, Andy Shih, Alexandre Robichaud, Paul-Vahe Cicek

**Affiliations:** 1 Microtechnologies Integration & Convergence Research Group, Université du Québec à Montréal, Montreal, QC H2X 3Y7, Canada; 2 Department of Electrical Engineering, École de Technologie Supérieure (ETS), Montreal, QC H3C 1K3, Canada; 3 Department of Applied Sciences, Université du Québec à Chicoutimi (UQAC), Chicoutimi, QC G7H 2B1, Canada

**Keywords:** lamb wave, mass sensing, microsensor, metalization ratio, CMOS, THD, acoustic microsensor

## Abstract

This paper presents the design and simulation of a mass sensitive Lamb wave microsensor with CMOS technology provided by SilTerra. In this work, the effects of the metalization ratio variation on the transmission gain, total harmonic distortion (THD), and two different resonant modes (around 66 MHz and 86 MHz) are shown. It has been found that the metalization ratio can be adjusted in order to obtain a compromise between transmission gain and sensitivity, depending on the design criteria. By adding a Si${}_{3}$N${}_{4}$ layer on top of the device, a five-fold improvement in transmission gain is reached. It was also shown that the transmission of the input differential IDT configuration is 20% more efficient than a single terminal. With this combination, the mass sensitivity is about 114 [cm${}^{2}$/gr].

## 1. Introduction

With ongoing improvements in microfabrication technologies, acoustic microsensors are becoming increasingly attractive for physical or chemical measurements within liquid and gas environments, such as relative humidity [1], temperature [2], pH [3], and pressure [4]. Cell detection and analysis is also a target of great interest within microfluidic systems [5,6,7,8]. In particular, acoustic mass sensing is a proven method to detect various chemical or biological analytes, and as such, can play a significant role in medical diagnosis [9,10]. For instance, Chang et al. used an acoustic microsensor for insulin detection, allowing the subsequent use of glycine-HCL to remove insulin for further testing [11]. Luo et al. developed a glucose biosensor using a multilayer Love-mode surface acoustic wave, achieving a sensitivity of 7.184 MHz/mM and an accuracy of 6.96 × 10^−3^ mM [12]. Moreover, acoustic sensors have been used to detect different DNA [13] and cancer cells [14]. Tigli et al. developed a surface acoustic wave (SAW) device using a gold layer to immobilize specific antibodies on its surface in order to detect a specific antigen that is a cancer biomarker [14]. Another application of mass sensing devices is to determine water quality by measuring and characterizing pH; biochemical oxygen demand; total organic carbon; and nitrate, nitrite, ammonia, chlorine, and fluoride concentrations [15,16]. Tamarind et al. presented a surface acoustic wave microfluidic chip with the ability to assess water quality on-site [15].

Acoustic microsensors are operated by applying an electrical signal to input interdigital electrodes (IDTs) situated on a piezoelectric material. The piezoelectric material transforms the electrical signal into mechanical waves [17], which travel within the substrate. At a certain distance from the input, output IDTs reconvert the mechanical waves into an electrical signal, but with a phase difference according to the distance traveled and the physical properties of the medium. Any mass added onto the piezoelectric sensing area between the input and output IDT will result in a proportional variation in the phase difference between input and output signals.

In order to detect the presence of an analyte within a liquid solution through mass sensing, immobilization must be achieved so as to selectively isolate the specific component of interest. In the context of biological sensing, where detection of an antigen is often of interest, antibodies can be affixed at the sensing zone using graphene [18], gold nanoparticles [19,20,21], or other suitable materials that are effective at retaining the specific antibody. When an antigen particle becomes attached to the immobilized antibodies on the sensing area, the variation in mass affects the resonance frequency of the sensor. This phenomenon can be harnessed to infer the amount of analyte that is circulating [22].

A critical factor in mass-sensitive acoustic devices is IDT geometry. The length, width, number, and shape of IDTs affect device behavior [23]. Skinner et al. studied the effect of IDT width on the efficiency of energy conversion of a SAW device [24]. Kuznetsova et al. evaluated the impacts of IDT finger length on acoustic plate wave synthesis [25]. There are also various configurations for applying actuation voltage to the IDT, leading to various electrical field distributions on the device. Zou et al. studied the effects of different electrode materials, transducer configurations, and electrode thicknesses on the coupling factor in an aluminum nitride (AlN) lamb wave resonator using the symmetric mode. As a result, they found a better coupling factor when using transducers on both sides of the piezoelectric device [26].

Acoustic microsensors can rely on different types of acoustic waves to measure or detect the presence of biological particles. For example, a lamb wave, which exhibits significant sensitivity to mass loading specifically within liquids [27], is an elastic wave that propagates along a thin layer membrane, whose thickness is less than or equivalent to the wavelength at play [9,28]. Kong et al. developed a lamb wave sensor to assess blood clot formation, in order to predict the risk of cardiovascular disease [29]. Lamb wave sensors can also be used for the detection of microparticles. Nam et al. implemented a lamb wave sensor that can detect the presence of nucleic acids in less than 30 min [30]. Lamb wave detection has been successfully demonstrated in environmental sensing applications such as humidity [31], temperature [32], and pressure [33] monitoring.

There are two main approaches for implementing these acoustic sensors. They can either be fabricated on a piezoelectric substrate to interface with a distinct electronic circuit or be integrated monolithically with complementary metal–oxide–semiconductor (CMOS) technology. With CMOS monolithic integration, the direct inclusion of integrated electronic circuits renders the whole system smaller and more compact, reduces parasitic capacitance, and has the potential to significantly lower costs at scale. Furthermore, assembly and packaging are simplified by eliminating the need for combining several heterogeneous chips with complex mounting or wirebonding schemes. References [34,35,36] present examples of heterogeneous sensor implementations. Tigli et al. implemented a CMOS integrated SAW device with a ZnO piezoelectric membrane for cancer biomarker detection with a frequency sensitivity of 8.704 pg/Hz [14]. In this paper, we propose a mass sensitive lamb wave microsensor designed with dual interdigital electrodes (IDT) for biological mass sensing, which can be integrated monolithically with CMOS SilTerra technology and its Si${}_{3}$N${}_{4}$ layer, which provides a fivefold improvement in transmission gain. The technology, aside from the inherent benefits provided by monolithic integration, features a very thin suspended piezoelectric layer of aluminum nitride (AlN), which promises superior sensitivity compared to thicker ones [37]. Furthermore, the top layer of silicon nitride (Si${}_{3}$N${}_{4}$) can be utilized to protect the device from direct contact with the fluidic environment [38], while also serving as an acoustic wave guiding layer to improving the transmission gain of the device. With the parameters of the selected technology for this work, lamb wave operation is the most appropriate with which to perform mass sensing. In order to optimize insertion loss and coupling factor, differential mode signals are investigated, and the impacts of IDT width on sensitivity and transmission gain. Total harmonic distortion (THD) is also studied to assess system linearity.

This article is divided into five sections: Section 2 provides a theoretical overview of lamb waves, IDTs, and the sensitivity metric; Section 3 details the parameters and specifics of the proposed device, and provides an overview of the simulation methodology; Section 4 presents and discusses the simulation results; Section 5 concludes.

## 2. Theoretical Background

Lamb wave velocity is defined by the waveguide material, and the *h*/λ ratio, in which *h* is the piezoelectric layer thickness and λ is the acoustic wavelength [37,39,40]. Both symmetric and antisymmetric modes can be generated in a lamb wave device [9]. The S0 mode, also called the extensional mode, is generated symmetrically, whereas the A0 mode, associated with flexural plate waves (FPWs), is antisymmetric. S0 and A0 sensitivities are the same for solid sensing, but there are some differences in liquid sensing [41]. The zero-order antisymmetric mode A0 is seen in devices operating in the range of 5 to 30 MHz [42] and exhibits low attenuation within liquids, which is essential for biological mass sensing. The shift in resonant frequency, Δ*f*, in response to a variation in analyte mass per unit area, Δ*m*, can be used as a means to detect the presence and measure the quantity of the analyte.

Mass sensitivity is defined as [37,43]:

(1) $$S_{m}=\frac{\Delta f}{\Delta m\cdot f_{s}}$$

where $f_{s}$ is the unloaded resonance frequency.

Radiation loss, which is a critical factor in lamb wave attenuation, is minimized in the A0 mode compared to the S0 mode, provided that its phase velocity is inferior to the velocity of a bulk acoustic wave in the liquid medium [44,45]. In the A0 mode, wave velocity can be controlled by the wavelength and the piezoelectric thickness; thus, a phase velocity lower than the nominal velocity of sound can be obtained, decreasing radiation losses in liquids. By increasing the piezoelectric layer’s thickness, the A0 and S0 mode waves converge with the Rayleigh mode [46].

Lin et al. showed that with an AlN-based symmetric mode lamb wave, by increasing *h*/λ to 1, the phase velocity increases to around 5.5 km/s [46]. In the present work, *h* is 1.3 µm and λ is 20 µm, yielding a phase velocity of around 0.8 km/s, corresponding to the *h*/λ vs. phase velocity curve presented by Lin et al. for the A0 mode.

**Figure 1:**
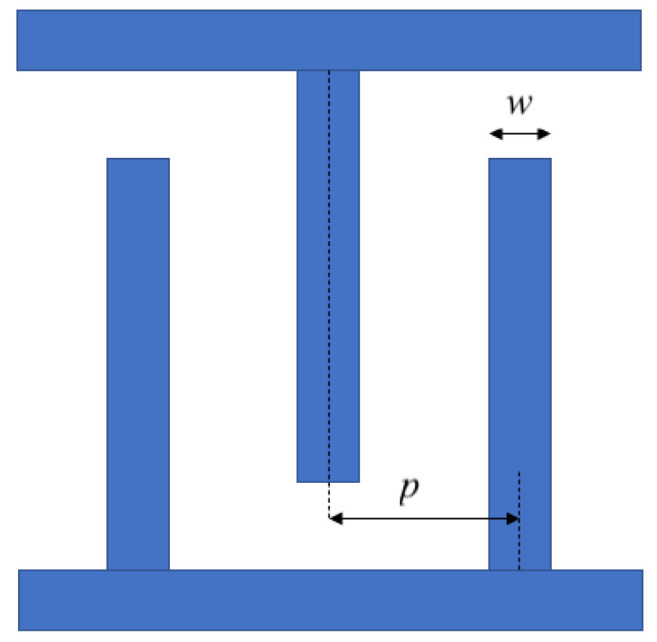
IDT with a metalization ratio of w/p.

IDT geometry affects certain properties of the device. IDT finger length and the number of electrodes determine electrical impedance: the lower the length, the higher the impedance [47]. Another important characteristic of IDT is the metalization ratio, η. It is defined as η=w/p where *w* is the finger width and *p* is the pitch (center-to-center) between each finger, as shown in Figure 1. η affects the coupling factor and the insertion loss [48]. The effects of η on sensitivity, transmission gain, and THD are also investigated; the results are presented in Section 3.

Skinner et al. studied the impact of metalization ratio on the efficiency of energy conversion for a SAW sensor, reporting optimal output energy for η of 0.74.

Within an acoustic waveguide, there are countless acoustic modes that can propagate [49]. Each mode has its specific shape and phase velocity ($v_{p}$). IDTs generate a force on the piezoelectric surface, producing a displacement in the piezoelectric resonator, which in turn can excite acoustic wave modes [50]. The mode whose displacement shape has more correlation (overlap integral [51]) with the displacement produced by the IDT will be excited with greater power. As a result, the greater the coupling efficiency, the greater the transmission gain will be. Varying the IDT metalization ratio induces a slightly different displacement shape. Depending on the shape of a given mode, its optimal IDT metalization ratio can vary. Considering that fundamental λ is dictated by IDT pitch, the resonant modes can appear at different frequencies as per $f=v_{p}/\lambda$.

In this study, the THD of the transmitted signal is also examined in order to assess the linearity of the system, or in other words, the extent to which varying the IDT metalization ratio introduces harmonic distortion. For a pure harmonic input signal at a given frequency, total harmonic distortion is defined as:

(2) $$\mathrm{THD}=\frac{\sqrt{V_{2}^{2}+V_{3}^{2}+V_{4}^{2}+\ldots }}{V_{1}}$$

where $V_{n}$ is the root mean square (RMS) voltage of the *n*th harmonic of the received signal.

## 3. System Overview and Simulation Methodology

**Figure 2:**
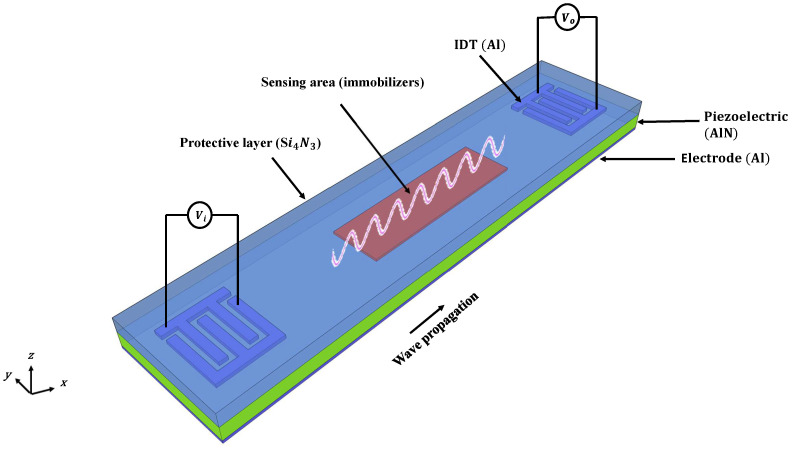
Three-dimensional schematic of the device showing different layer order, IDT locations, and the wave propagation direction.

The two-port device developed in this work through the SilTerra technology is schematized in Figure 2. It consists of a thin layer of aluminum beneath 1.3 µm of aluminum nitride (AlN), the piezoelectric material, covered by 1.5 µm of Si${}_{3}$N${}_{4}$, the protection and waveguide material. The separation between the transmitter and receiver IDT acts as the delay line, enclosing the sensing area with the immobilizers that were explained in the previous section.

**Figure 3:**
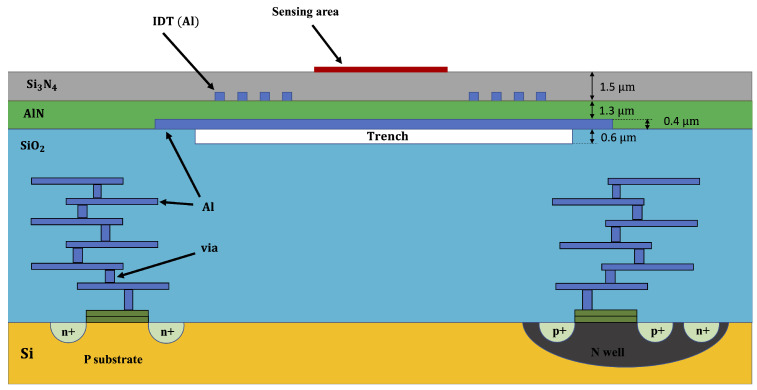
Cross-section of the Lamb wave microsensor realized with the CMOS Silterra technology.

The SilTerra technology provides a suspended AlN piezoelectric layer directly above a conventional 130 nm CMOS semiconductor process, as illustrated in Figure 3. The technology features a thin layer of AlN, which helps improve sensitivity [37] and a Si${}_{3}$N${}_{4}$ layer for protection and wave guiding.

**Figure 4:**
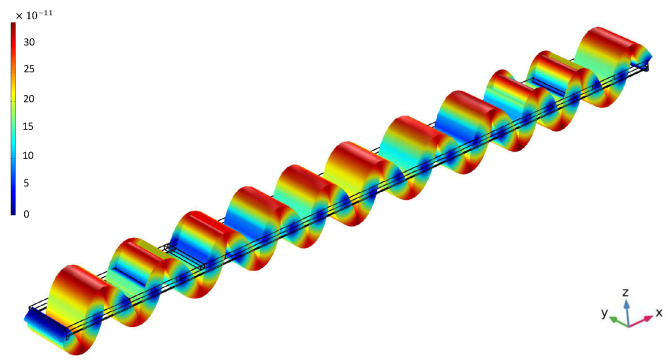
Time-domain simulation of the lamb wave illustrating the wave propagation and the total displacement of each part of the device at 216 ns with an input signal frequency of 86 MHz and metalization ratio of 85%.

In this work, COMSOL Multiphysics 6.0, Stockholm, Sweden, was used to perform finite-element method (FEM) simulations. An sinusoidal electrical signal was applied to the input IDT and transformed into mechanical waves through the piezoelectric layer (as shown in Figure 4, 200 ns after voltage application). The resulting induced electrical signal at the output IDT was monitored and analyzed to design a sensitive mass sensor.

One of the methods to detect a variation of mass in acoustic sensors is to measure the device’s resonance frequency before and after mass loading. Based on (1), the greater the frequency shift, the greater the sensitivity [43]. The physical properties of AlN used in the simulation are listed in Table 1.

**Figure 5:**
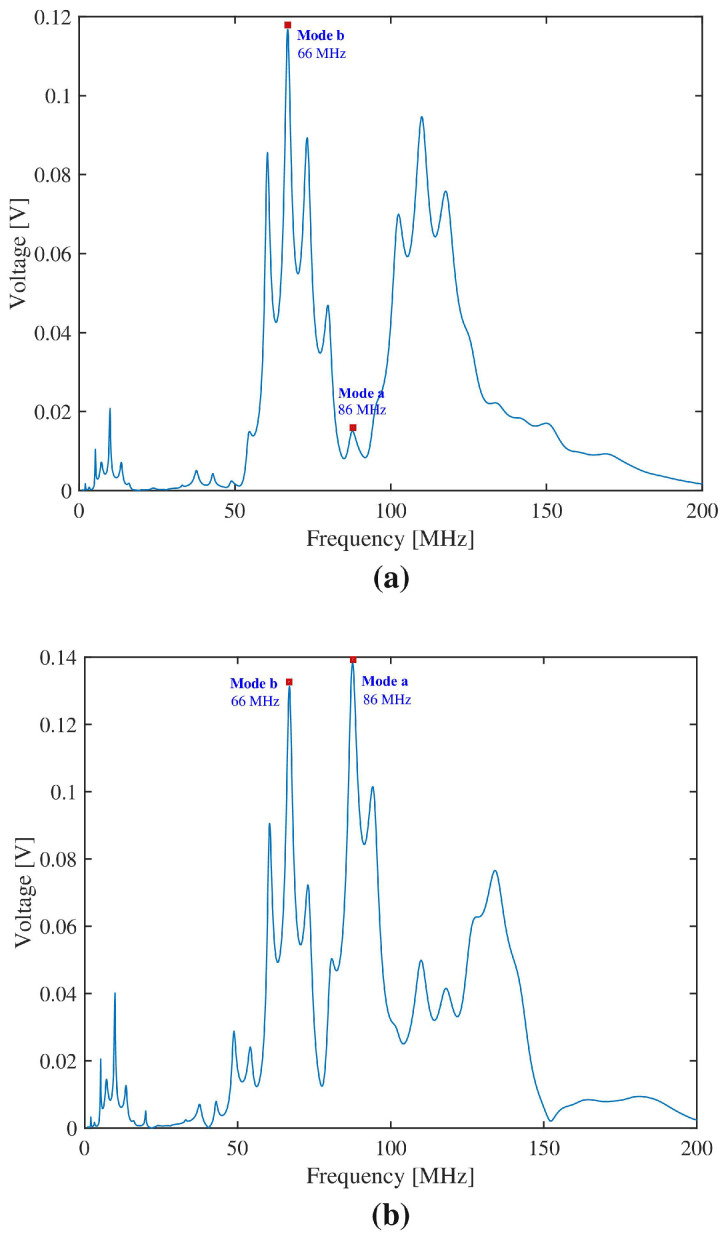

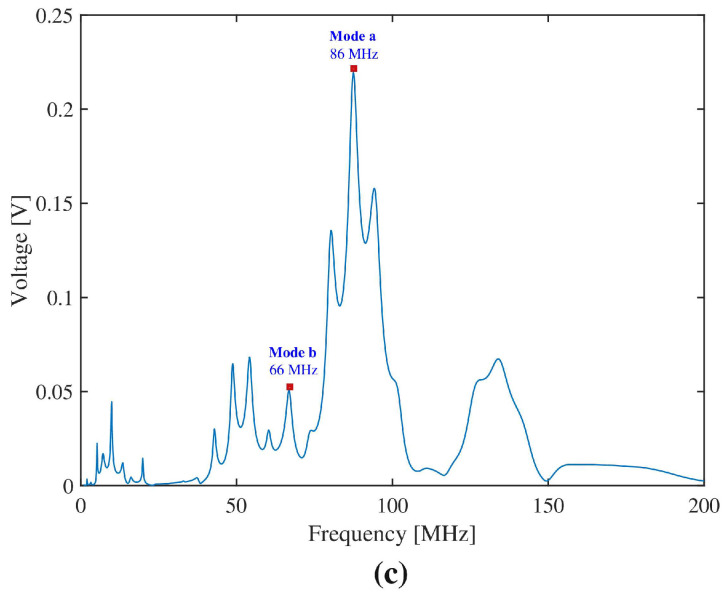
Mode a and mode b shown in output frequency spectrum for metalization ratios of (**a**) 10%, (**b**) 50%, and (**c**) 85%.

All FEM simulations were performed using COMSOL Multiphysics and analyzed in Mathworks MATLAB R2022a, Natick, MA, USA. Importantly, all layer physical properties and dimensions followed the specifications, guidelines, and design rules of the SilTerra technology, in order to ensure that the CMOS-compatible acoustic sensor design would be implementable. The input IDTs were defined as terminals with a sinusoidal signal of $5\sin\left(2\pi f_{r}t\right)$. In order to choose the excitation frequency $f_{r}$, a frequency study was first performed to determine the resonance frequency peaks for the design under consideration, as shown in Figure 5. The wave modes corresponding to these resonance frequency peaks were determined by analyzing their respective wave deformation shapes. It can be observed that the localization of the dominant frequency peak changes when the metalization ratio passes about 50%. For a metalization ratio superior or equal to 50%, the dominant frequency is located at 86 MHz and is labeled *mode a*. For a metalization ratio inferior to 50%, the dominant frequency peak is located at 66 MHz and is labeled *mode b*. Both a and b modes behave similarly to A0 lamb waves but for different phase velocities. The phase velocity for *mode a* at a metalization ratio of 85% was determined to be 2.013 km/s, whereas it was found to be 1.672 km/s for *mode b* at a metalization ratio of 40%. It is posited that the existence of these two A0-like modes can be explained by the varying mode shape deformations induced by the different metalization ratios.

**Figure 6:**
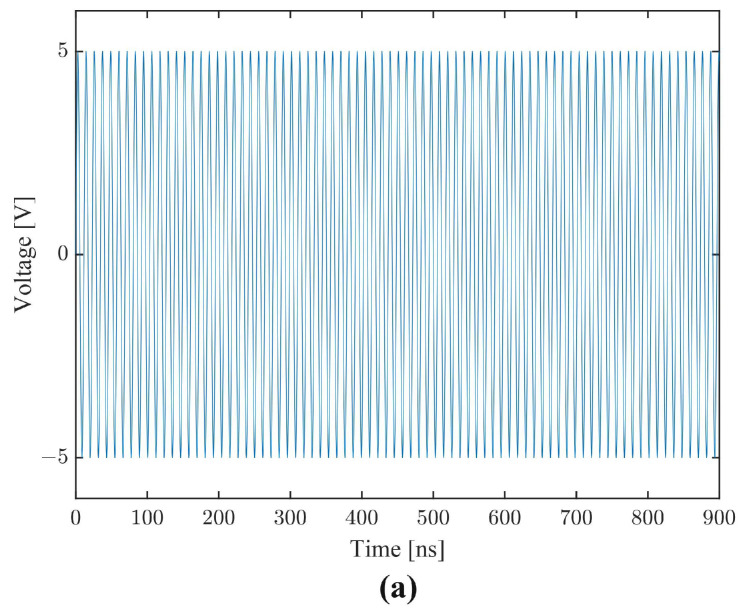

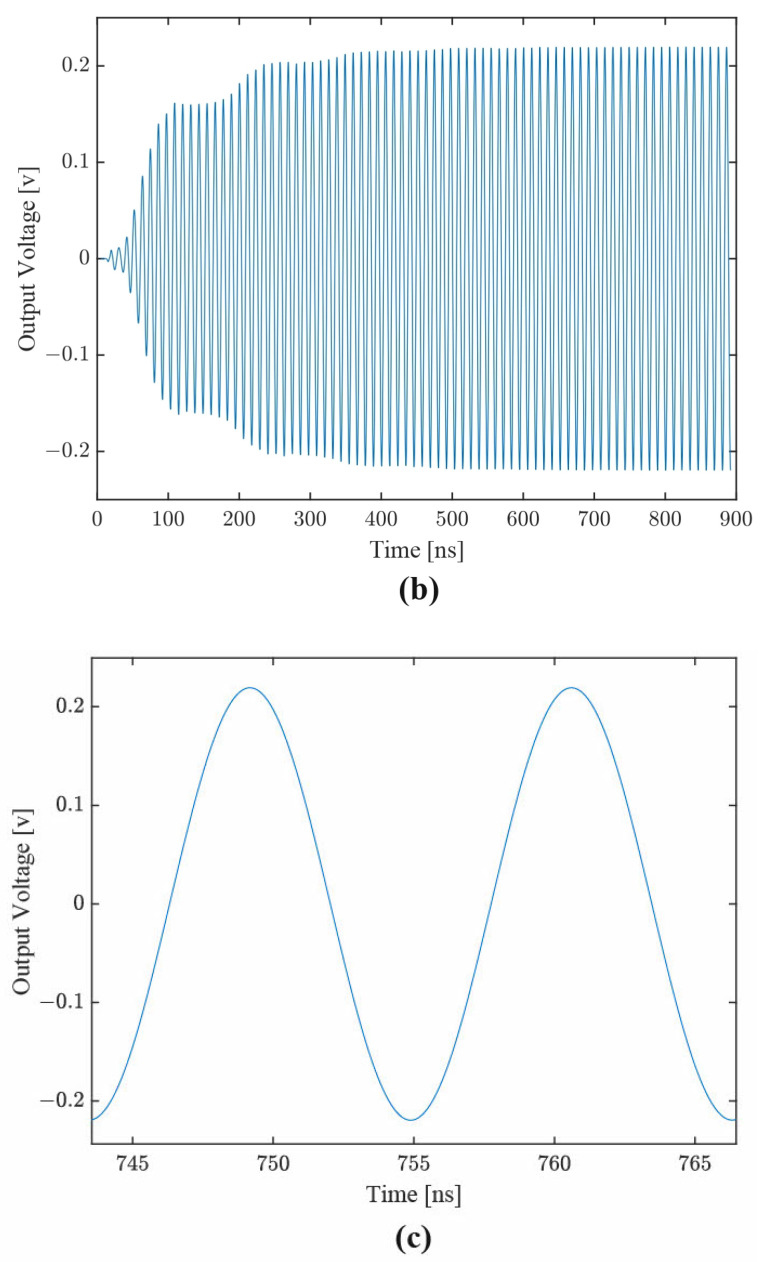
Time domain simulation waveforms for IDT metalization ratio of 85%: (**a**) harmonic signal with amplitude of 5 V applied to input IDT starting at time t = 0; (**b**) received signal at output IDT; (**c**) close-up of two periods of received signal from 745 to 765 ns.

In order to configure the simulation model to accurately represent reality, two periodic boundary conditions are used on both ends of the device parallel to wave propagation, along with low reflecting boundaries for both ends orthogonal to wave propagation, in order to eliminate any unrepresentative wave reflections that might behave destructively. As shown in Figure 6, it takes about 40 ns for the acoustic signal to reach the output IDT, but about 350 ns for it to stabilize. To assess mass sensitivity in COMSOL without varying other device conditions, a thin layer of immobilizer PMMA is placed above the Si${}_{3}$N${}_{4}$. To simulate the loading of additional mass, the density of the PMMA is correspondingly increased. Any increase in mass density affects the velocity of the lamb wave and alters the resonance frequency and phase shift of the device. As a result, mass sensitivity can be inferred.

**Figure 7:**
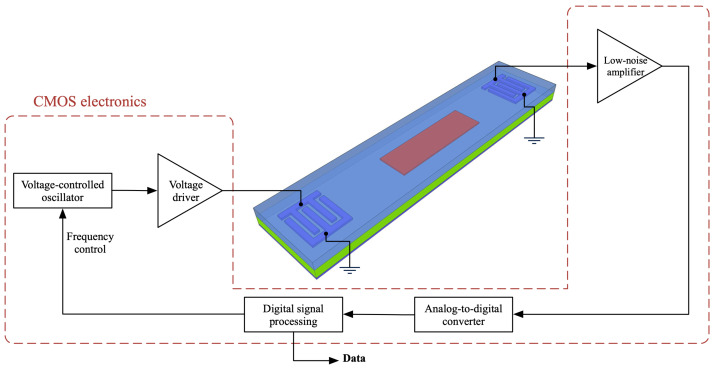
Block-level diagram of a possible integrated detection system.

The use of a technology providing monolithic integration of acoustic and CMOS semiconductor devices creates the possibility of a full chip-scale system, such as the one suggested in Figure 7. In this setup, a harmonic signal is generated at a specific frequency, as defined by the digital signal processing (DSP) unit, then amplified and fed to the input IDT. A harmonic signal at the same frequency is received and amplified at the output IDT, digitized, and processed by the DSP. By sweeping the excitation frequency, DSP can determine the system’s resonance frequency, hence the deposited mass on the sensor.

**Figure 8:**
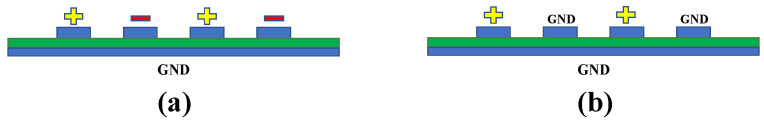
Illustration of the (**a**) differential and (**b**) single-ended electrode configurations, highlighting positive, negative, and ground (GND) signals.

In Figure 8, two alternate methods are used to apply the electrical signals to the input IDT. The first is the differential approach in which IDT are alternately connected to the negative and positive phases of a sinusoidal signal. The second is the single-ended method in which IDT are alternately connected to a single phase of a sinusoidal signal and ground. In both topologies, the bottom plane is connected to the ground.

Lin et al. have presented equivalent circuit models for different methods of applying signals for single-ended configurations, and concluded that grounding the bottom plane increases static capacitance compared to a floating plane, which improves the coupling coefficient [46].

To determine THD, a frequency simulation was first performed in COMSOL Multiphysics to find the resonance frequency of the current device configuration. Subsequently, using an input signal at the determined frequency, a 1000 ns time-dependent simulation was run with a time step of 0.01 ns. Using this time-series output voltage data, THD was calculated using MATLAB.

## 4. Results and Discussion

In this part, the effects of metalization ratio on different characteristics of the device are explored. In particular, its effects on gain, sensitivity, and THD in different configurations of the device are assessed. These include simulations with and without the Si${}_{3}$N${}_{4}$ layer and single-ended versus differential voltage stimulation.

**Figure 9:**
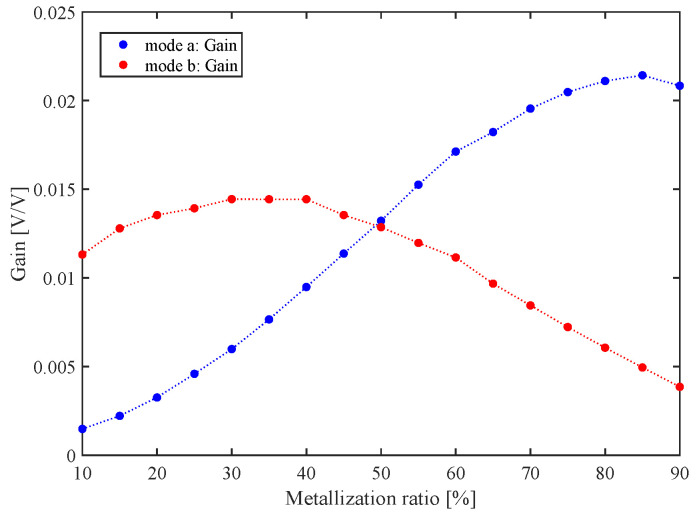
Transmission gain with respect to IDT metalization ratio.

The transmission gain of this device is shown in Figure 9. When driving the device at the resonance frequency of mode a (about 86 MHz), transmission gain is maximal at 0.0214 for a metalization ratio of 85%. When exciting mode b, with a resonance frequency of about 66 MHz, the gain reaches its maximum of 0.0144 for a ratio between 30% and 40%. Figure 9 suggests that mode a is optimal for a metalization ratio above 50%, whereas mode b is optimal for a metalization ratio below 50%, where the two transmission gain curves cross each other.

**Figure 10:**
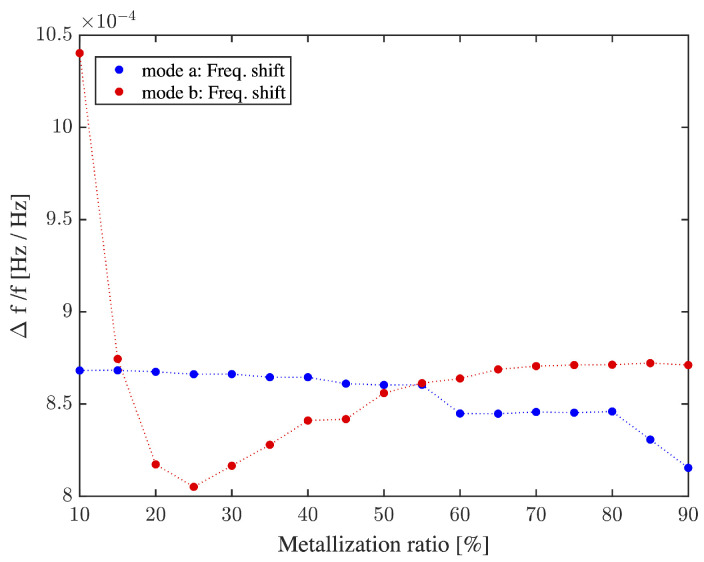
Sensitivity of the device depending on IDT metalization ratio in response to a 100 kg/m${}^{3}$ increase in PMMA density.

Figure 10 presents the variations in frequency sensitivity compared to mass variation, as the IDT metalization ratio is varied from 10% to 90%. Both deformation modes explained in Section 2 are examined. For mode b, mass sensitivity is nearly constant for a metalization ratio ranging from 10% to 90%. In mode a, mass sensitivity is at its maximum for an IDT metalization ratio of 10%, but is associated with a low transmission gain which would make it more challenging to discern the output signal from noise. However, for this mode, transmission gain gradually increases from a metalization ratio of 25% up to 70%, and then starts to plateau. As shown in Figure 10, considering the tradeoff between gain and sensitivity, the sensitivity remains mostly constant at about 114 cm${}^{2}$/g with 10% to 90% metalization ratios. As a point of reference, assuming a minimum detectable frequency variation of 1 Hz for the system, its sensitivity would allow a detection resolution of 1.2 ng, or the equivalent of about a hundred bacteria of typical weight [52].

Conventionally, metalization ratio is usually fixed at 50% which represents standard bidirectional IDT [9,44,53,54]. However, in this work, we show that it is possible to achieve a superior tradeoff between insertion loss and mass sensitivity by adjusting the metalization ratio for a given deformation mode, according to the specification priorities. For instance, in mode a, mass sensitivity is maximal for a metalization ratio of 10%, but with poor transmission gain. However, for the same mode, a metalization ratio of 80% provides maximal transmission gain, also with reasonable mass sensitivity. As for mode b, mass sensitivity is maximal for a metalization ratio below 55%, whereas transmission gain reaches its peak at about 35%. In this case, a metalization ratio of 80% is obviously optimal.

**Figure 11:**
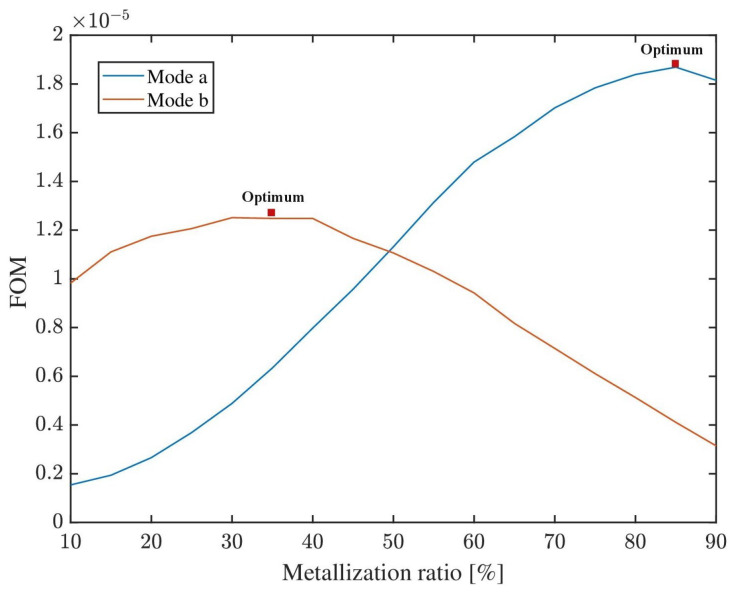
Arbitrary FOM shows the ability to optimize by means of metalization ratio.

According to the requirements of the designer, it is reasonable to establish a figure of merit (FOM) in order to optimize the selection of the metalization ratio. As a generic example, the following (plotted in Figure 11) attributes equal value to transmission gain and mass sensitivity:

(3) FOM=TransmissionGain×MassSensitivity

Although constituting a very simple example, the FOM could be finely adjusted according to design specifications and priorities, and could even incorporate additional performance metrics of interest (e.g., THD, power consumption, size). Figure 11 illustrates the ability to select an optimal metalization ratio in order to maximize a chosen FOM.

**Figure 12:**
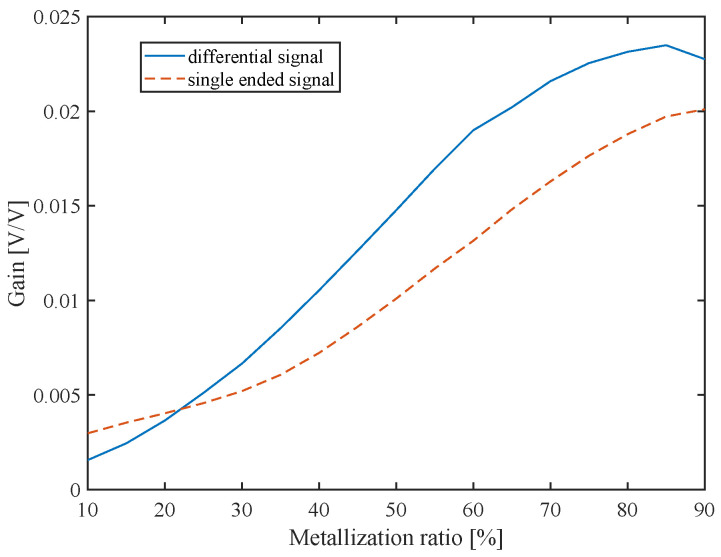
Device gain of single-ended and differential mode for metalization ratios ranging from 10% to 90% operating in mode a.

All subsequent results were obtained using an input frequency of 86 MHz (mode a). In Section 2, two methods of applying the input signal were presented: single-ended (typical) and differential. To perform a fair comparison, the amplitude of the input signal in the single-ended configuration was doubled (10 V) compared to the differential (5 V). As shown in Figure 12, the differential configuration moderately improved transmission gain, reaching a maximum of 0.0235 for a metalization ratio of 85%. In the single-ended method, the maximum gain was 0.02 for a metalization ratio of 90%.

**Figure 13:**
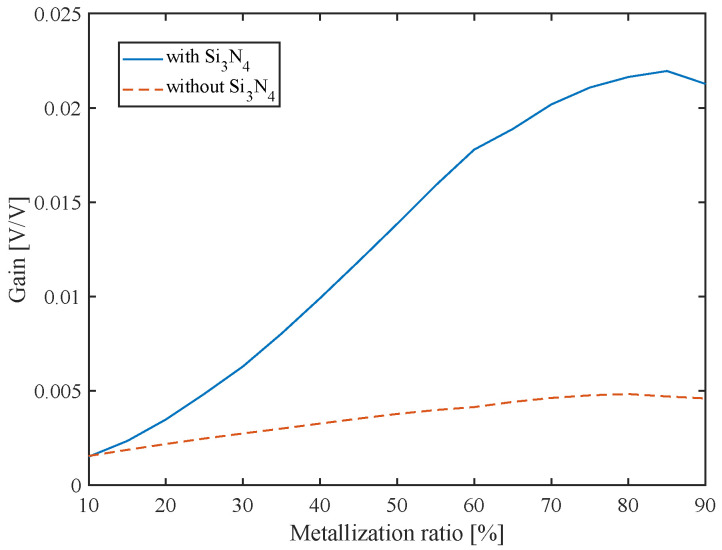
The effect of the Si${}_{3}$N${}_{4}$ layer on the device’s gain for metalization ratios ranging from 10% to 90% operating in mode a.

The physical properties of all layers play major roles in acoustic sensing: a small thickness variation in the stack of materials could affect insertion loss (transmission gain) and sensitivity. Figure 13 shows that the Si${}_{3}$N${}_{4}$ layer has a significant effect on transmission gain, and hence on acoustic coupling. The acoustic velocity of Si${}_{3}$N${}_{4}$ is lower than that of the piezoelectric layer (AlN), which allows Si${}_{3}$N${}_{4}$ to behave as a guiding layer that concentrates the acoustic energy in the active device [55]. Without this guiding layer, the maximum transmission gain is 0.0048, whereas, with Si${}_{3}$N${}_{4}$ present, the maximum transmission gain is improved to 0.0235, i.e., a 4.8 times increase.

**Figure 14:**
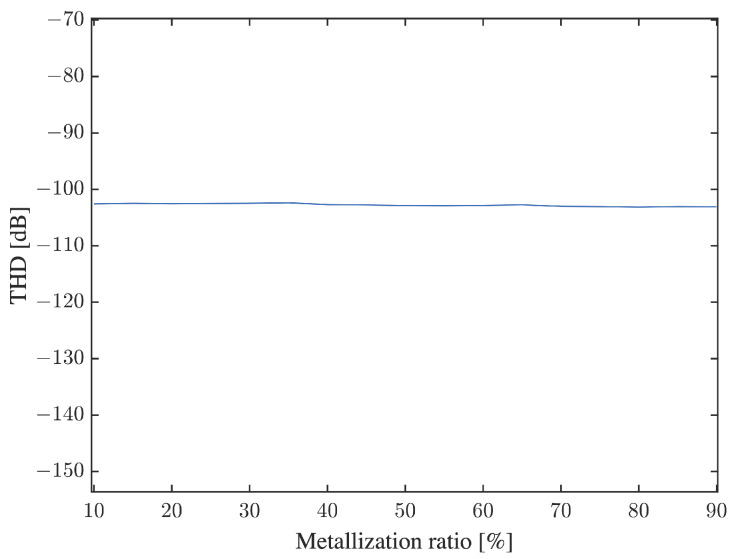
The effect of IDT’s metalization ratio on the device’s THD operating in mode a.

When adjusting the IDT metalization ratio to optimize sensor characteristics such as mass sensitivity and transmission gain, a serious worry is that any geometrical asymmetry might affect the shape of the generated waveform, and thus adversely impact system linearity. These concerns are alleviated in Figure 14, which demonstrates that THD remains stable across metalization ratios and inferior to a very low value of −108 dB, regardless of metalization ratio.

THD was calculated by applying Equation (2) to the stabilized portion of the output time series of the simulated device. For example, in Figure 6, outputs between 400 and 900 ns was used. Confirming the linearity of the system, Figure 6b shows a smooth harmonic output exempt of any visible distortion.

Table 2 presents the performance of the design of this work, in relation to acoustic sensors from the literature. Specifically, mass sensitivity ($S_{m}$) and insertion loss (IL) are compared. Insertion loss can be calculated from the transmission gain using

(4) $$\mathrm{Insertion}\;\mathrm{loss}\;\left(\mathrm{dB}\right)=10\log_{10}\frac{|V_{i}{|}^{2}}{|V_{o}{|}^{2}}=20\log_{10}\frac{|V_{i}|}{|V_{o}|}=20\log_{10}\frac{1}{\mathrm{gain}}.$$

With FOM chosen as the ratio between mass sensitivity and insertion loss, two versions of the design of this work for different IDT metalization ratios were evaluated: one minimizing insertion loss and the other maximizing mass sensitivity. Both proposed designs were found to be reasonably competitive with the state of the art, despite the use of non-optimized commercial technology with set material properties and thicknesses.

## 5. Conclusions

This work presented the design and simulation of a mass sensitive lamb wave microsensor in the CMOS-based technology provided by SilTerra. It was shown that the Si${}_{3}$N${}_{4}$ layer present in the technology could provide a fivefold improvement in transmission gain by serving as a guiding layer. The designed devices were analyzed in two different resonant modes (around 66 and 86 MHz). It was established that the metalization ratio can be adjusted in order to achieve an optimal tradeoff between transmission gain and sensitivity, depending on design criteria. It was also determined that the input IDT differential configuration is marginally more efficient than the single-ended one, with a 20% greater transmission gain. Worries about metalization ratio having any influence on output signal THD were unfounded, with very low levels (−100 dB) for all ratios. Although FEM simulation results can admittedly differ from practical results due to material parameters, mesh structure, and physics simplifications, this work was able to present a general methodology for acoustic wave sensor optimization based on structural topology. Although results may vary according to technology and device type, the general approach remains valid and worthwhile. Physical devices, currently in the fabrication pipeline, will be tested as soon as possible in order to validate this work’s conclusions.

## Figures and Tables

**Table 1:** AlN physical properties used in this work’s simulations [26].

	Symbol	AlN	Unit
Stiffness constants	$C_{11}$	345	$10^{9}$ [N/m${}^{2}$]
	$C_{12}$	125	
	$C_{13}$	120	
	$C_{33}$	395	
	$C_{44}$	118	
	$C_{66}$	110	
Dielectric constants	$\varepsilon_{11}$	8.0	$10^{-}{}^{1}{}^{1}$ [F/N]
	$\varepsilon_{33}$	9.5	
Piezo constants	$e_{15}$	−0.48	[C/m${}^{2}$]
	$e_{31}$	−0.58	
	$e_{33}$	1.55	
Mass density	ρ	3260	[Kg/m${}^{3}$]

**Table 2:** Comparison of acoustic wave sensors’ performance.

Biosensor Type	$S_{m}$ [cm${}^{2}$/g]	IL [dB]	FOM ($S_{m}$/IL)	Piezoelectric Material	Reference
SAW	2.6–121.7	25–55	1.196	ZnO	[56]
SAW	-	52.5–53.25	-	ZnO/quartz	[56]
SAW	70	-	-	ZnO/LiTaO_3_	[57]
FPW	60.16–70.06	36.04	1.944	ZnO	[58]
LW	160–240	-	-	AlN	[59]
LW	91.65	18.53	4.946	ZnO	[60]
LW(A0)	175.8	45	3.907	AlN	[61]
LW(A0 & S0)	174–272	20–38	13.6	GaN	[62]
**LW (Min IL)**	114	26.74	4.263	AlN	This work
**LW (Max $S_{m}$)**	140	66.02	2.12	AlN	This work

## Data Availability

Not applicable.
